# Supplementary material for: Influence of Anode Immersion Speed on Current and Power in Plasma Electrolytic Polishing
Source: Micromachines (Basel). 2024 Jun 14;15(6):783. doi: 10.3390/mi15060783 (PMC11205743; doi:10.3390/mi15060783)
Supplement: Supplementary file 1 [file micromachines-15-00783-s001.zip › Supplement_file_Valentincic_etal.pdf]

Supplementary material

Influence of Anode Immersion Speed on Current and Power in Plasma Electrolytic Polishing

Joško Valentinčič [1,*], Henning Zeidler [2], Toni Boettger [2] and Marko Jerman [1]

[1] Faculty of Mechanical Engineering, University of Ljubljana, Aškerčeva 6, 1000 Ljubljana, Slovenia; marko.jerman@fs.uni-lj.si
[2] Chair Additive Manufacturing, Technische Universität Bergakademie Freiberg, Agricolastrasse 1, 09599 Freiberg, Germany; henning.zeidler@imkf.tu-freiberg.de (H.Z.); toni.boettger@imkf.tu-freiberg.de (T.B.)
* Correspondence: jv@fs.uni-lj.si; Tel.: +386-1-4771-730

S1: Experimental data / Data related to signal acquisition

The voltage used in experiments was constant and fixed to 350 V. Total travel distance of the anode was 330 mm. In order to successfully accelerate the anode to the required speed before touching the electrolyte, the distance from the top position of the anode in z axis to the electrolyte surface was set to the maximum value, i.e. 215 mm.

The acquisition parameters are listed in Table S1 together with Savitzky-Golay filtering parameters. The value *filter* was set according to the sampling rate and calculated within a Matlab script: *n*=round(20e-6***sampleRate*);.
It represents a number of point to the each side of the reference point that are taken into account to calculate the new, filtered value of the reference point. Hence, 20 µs on both sides of the reference point was considered, at each sampling rate used. The degree of the least squares polynomial is denoted as *M* in Table S1 and it was 2 for all immesion speeds.

Table S1: Acquisition and Savitzky-Golay smoothing filter parameters

v (mm s^{-1})	No. of samples acquired	Current amplification (A/V)	Sampling rate (MHz)	Savitzky-Golay filter	
				n	*M*
5		5	31.5	630	2
20		50	41.5	830	2
100		5	125	2500	2
200		5	125	2500	2
300		50	125	2500	2
400		50	1.98	40	2
500		50	1.98	40	2
touched		50	25	500	2
submerged		50	25	500	2

The selected sampling rates require additional explanation. The experiments started with the velocity 400 mm s^{-1} and continued with 500 mm s^{-1}. Here, relatively low sampling rate was selected. The

higher sampling rates were used for higher immersion speeds to better describe the phenomena when the anode gets in contact with the electrolyte.

The sampling rates are lower for immersion speeds 400 mm s^{-1} and 500 mm s^{-1}. Since results are in agreement with other immersion speeds, the results do not depend on the sampling rate in this range of sampling rates.

S2: Part of original and filtered signal (v = 400 mm s^{-1})

A Savitzky-Golay smoothing filter was used for filtering the voltage and current signal. A second-degree polynomial function was used and interval of 20 µs before and after the observed sample on the signal was the used. The filtered voltage and current signals were used to calculate the power signal.

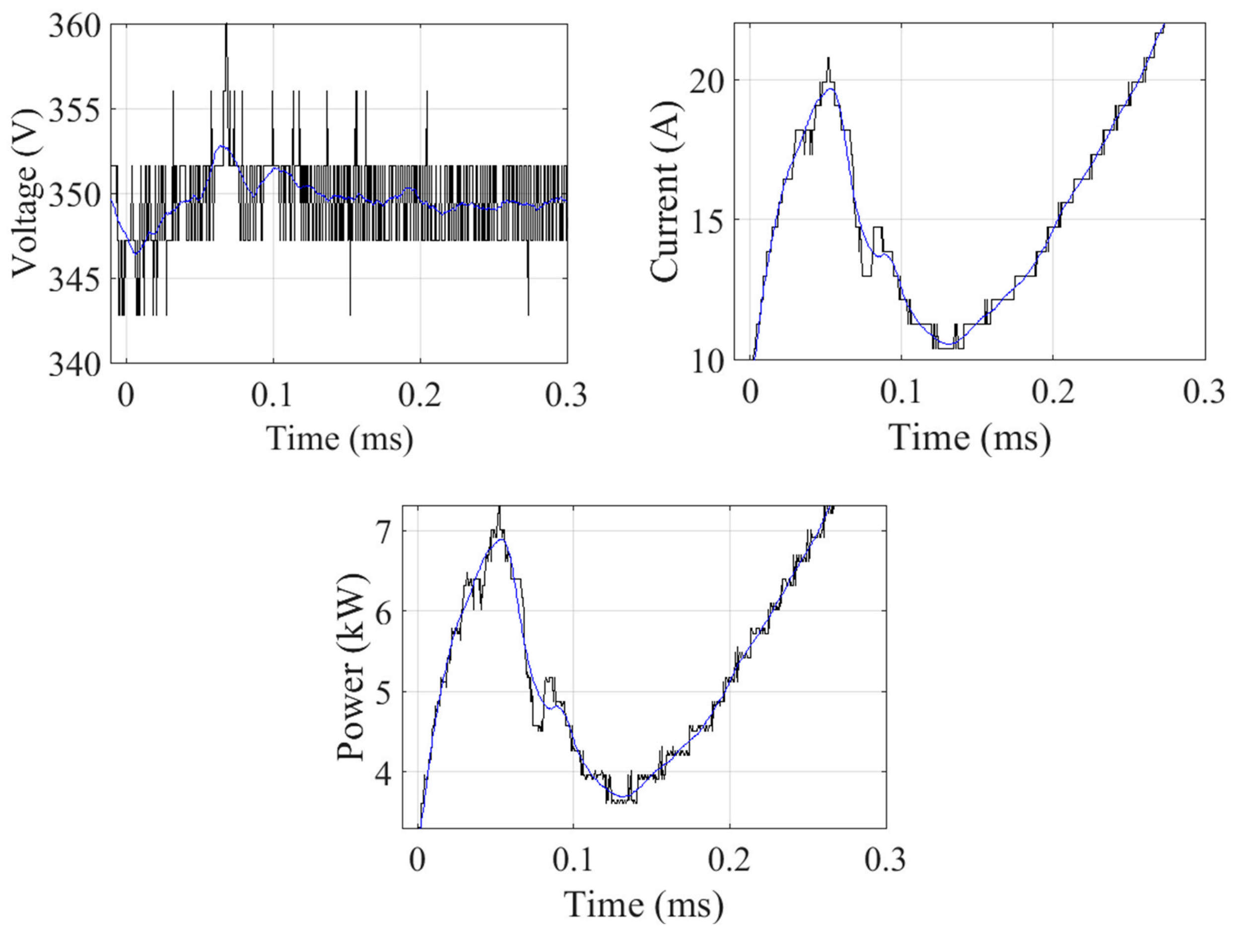

S3: Waveforms acquired when process was initiated with fully submerged anode and when the anode is touching the electrolyte

Three peaks are always present during the process initiation when the anode is fully submerged.

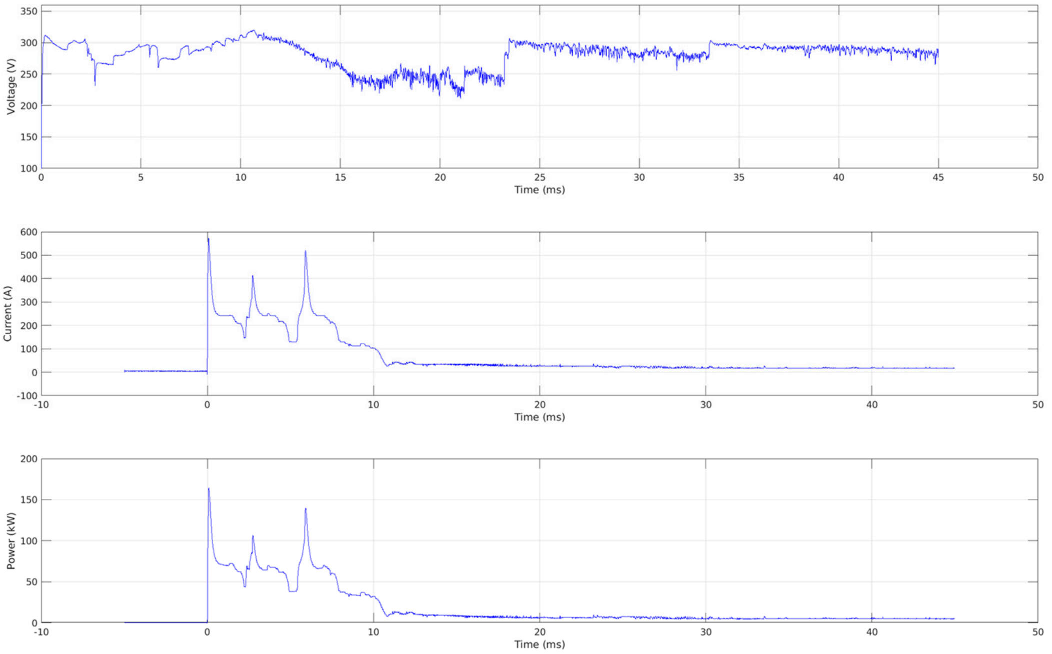

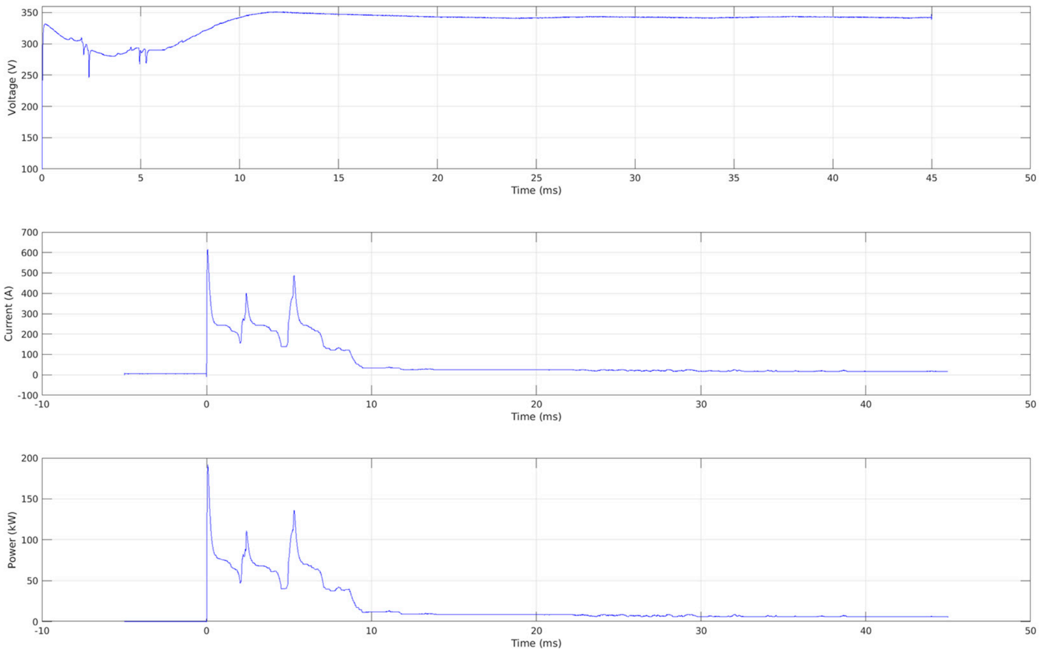

The same repeatability is observed during the process initiation when the anode is touching the electrolyte.

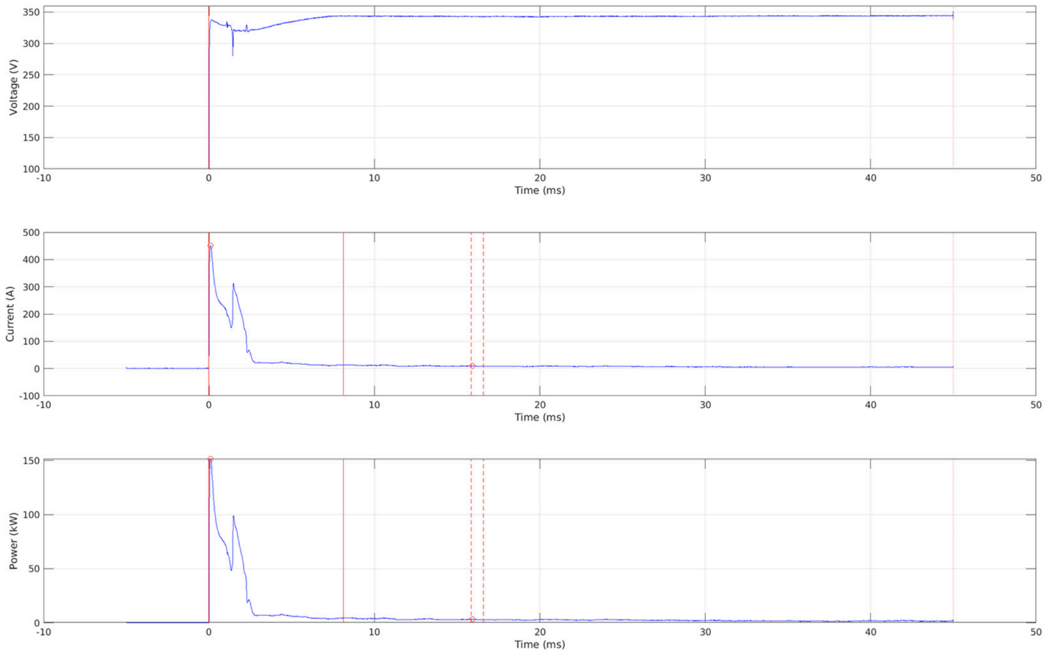

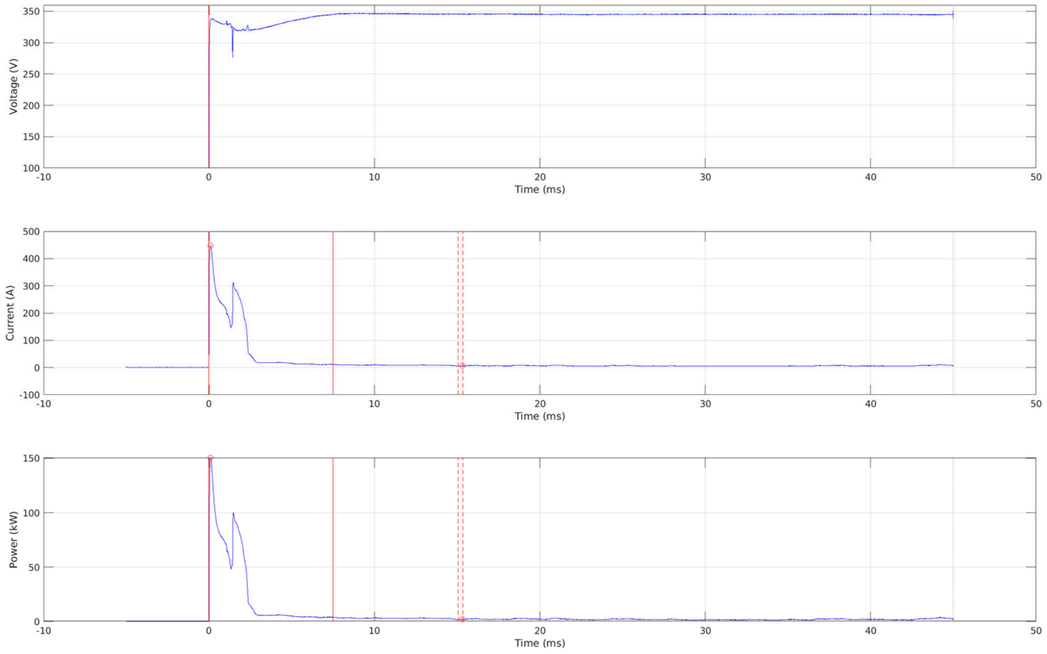

S4: Waveforms acquired when immersion speed was 400 mm s^{-1}

A high level of repeatability is noticed.

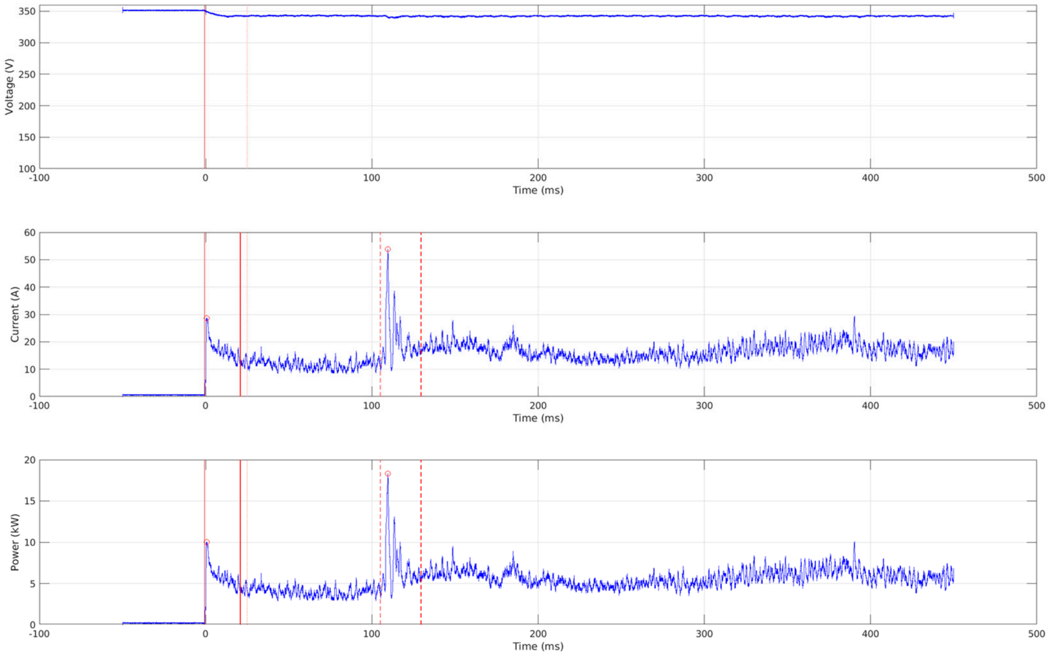

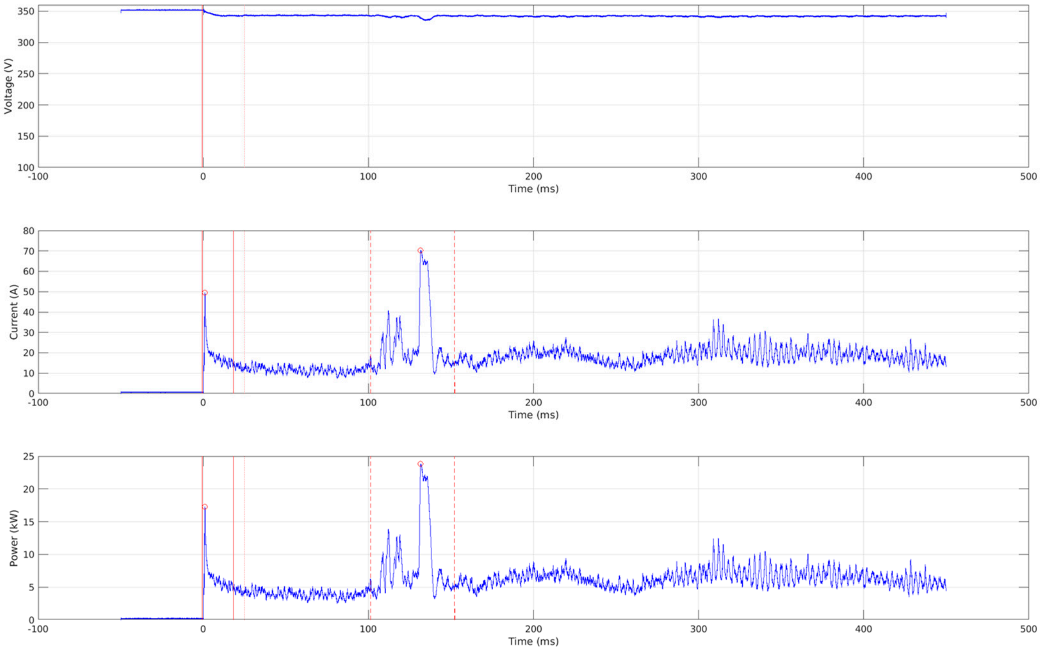

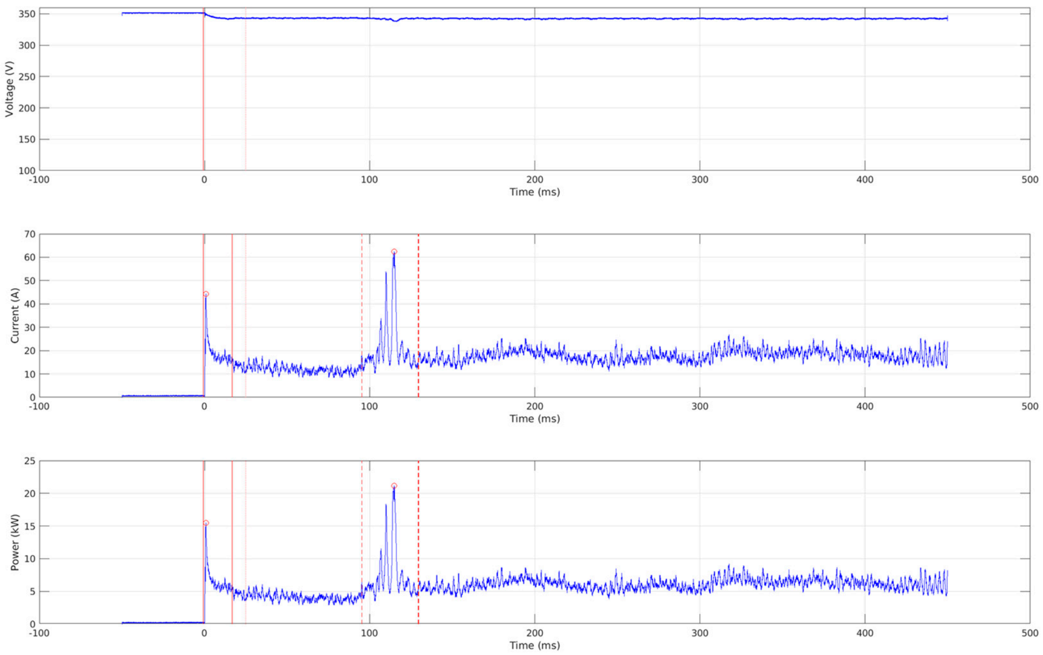

Voltage (V)
Current (A)
Power (kW)
Time (ms)

S5: Waveforms acquired at all immersion speeds

v=5 mm s^{-1}

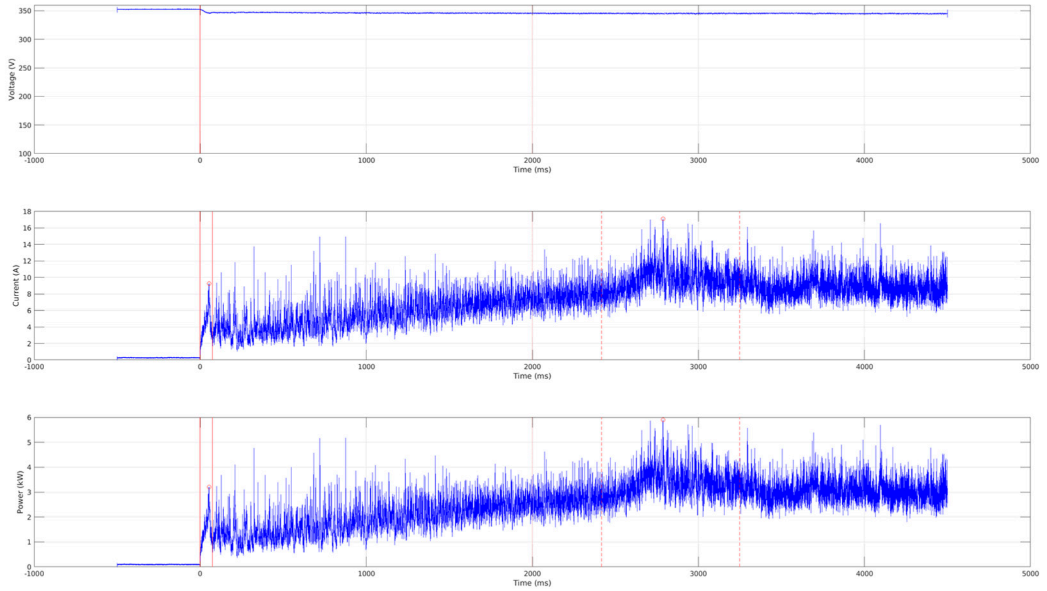

v=20 mm s^{-1}

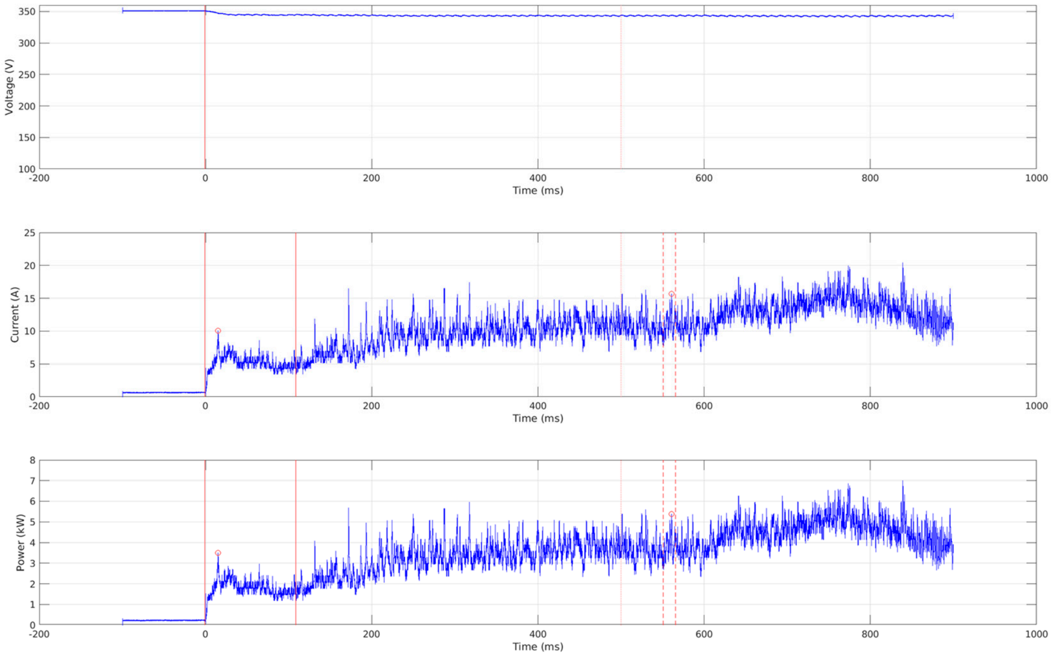

v=100 mm s^{-1}

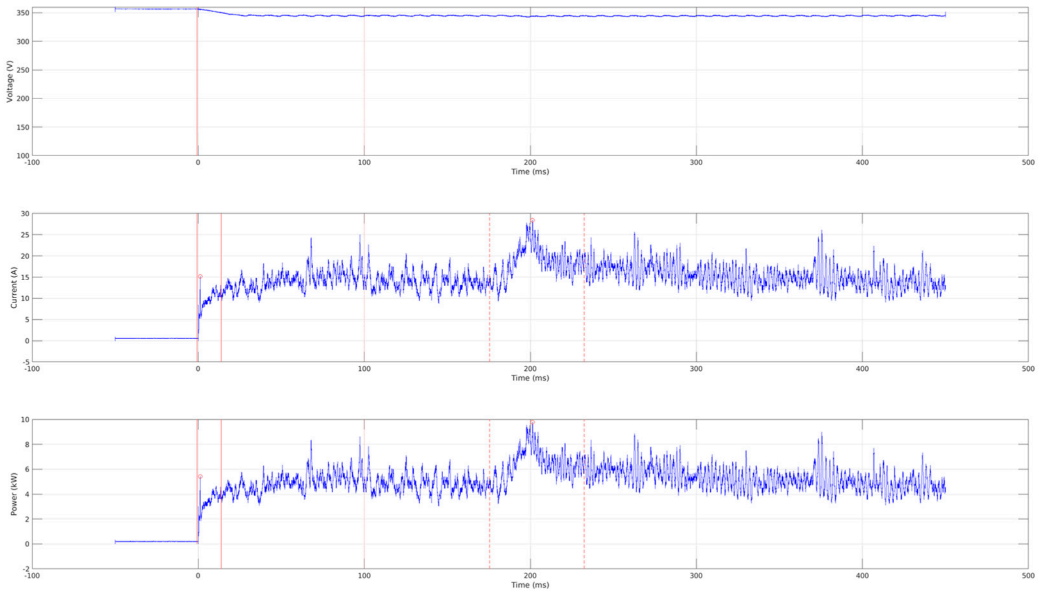

v=200 mm s^{-1}

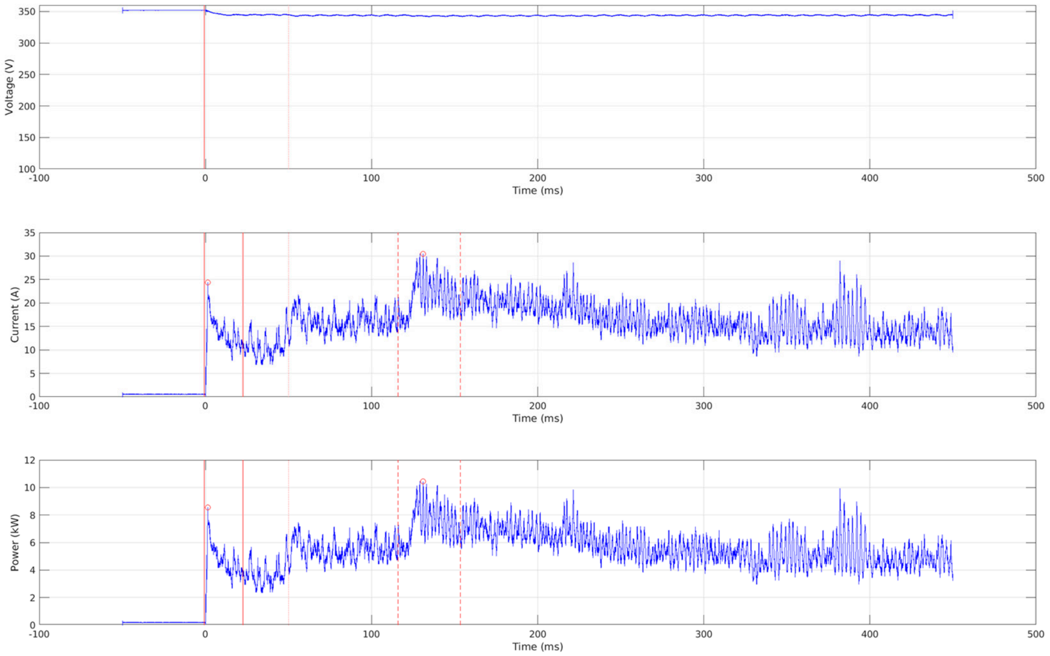

v=300 mm s^{-1}

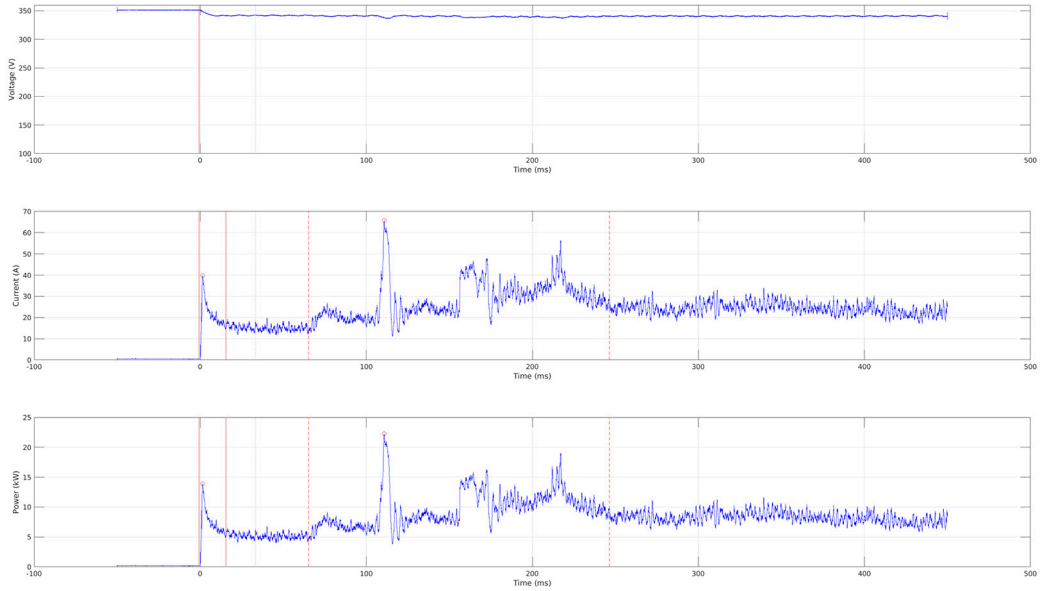

v=400 mm s^{-1}

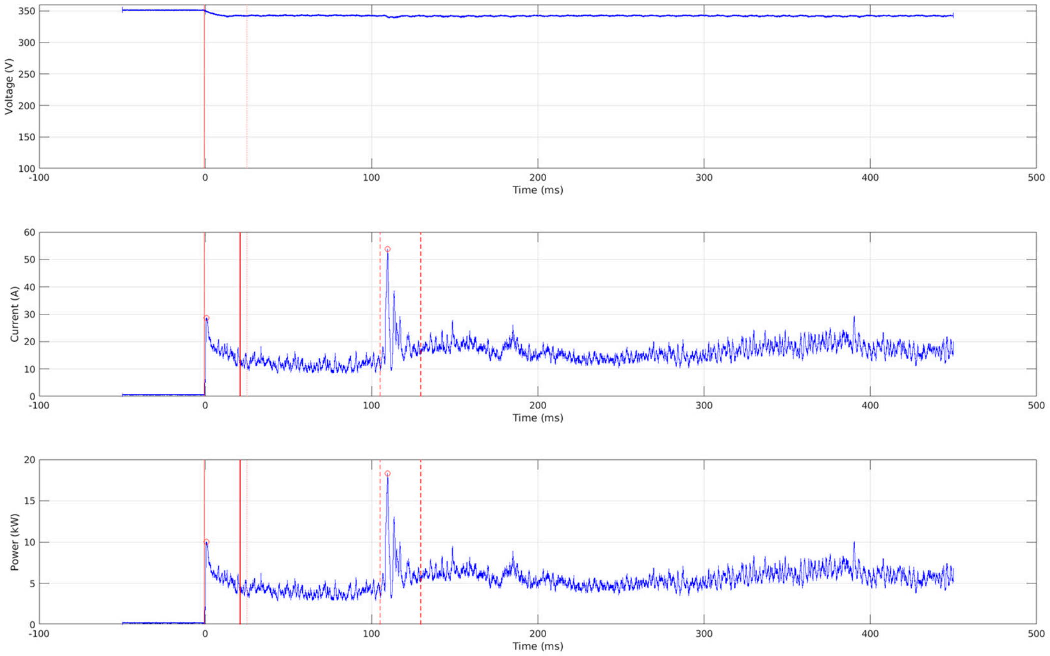

v=500 mm s^{-1}

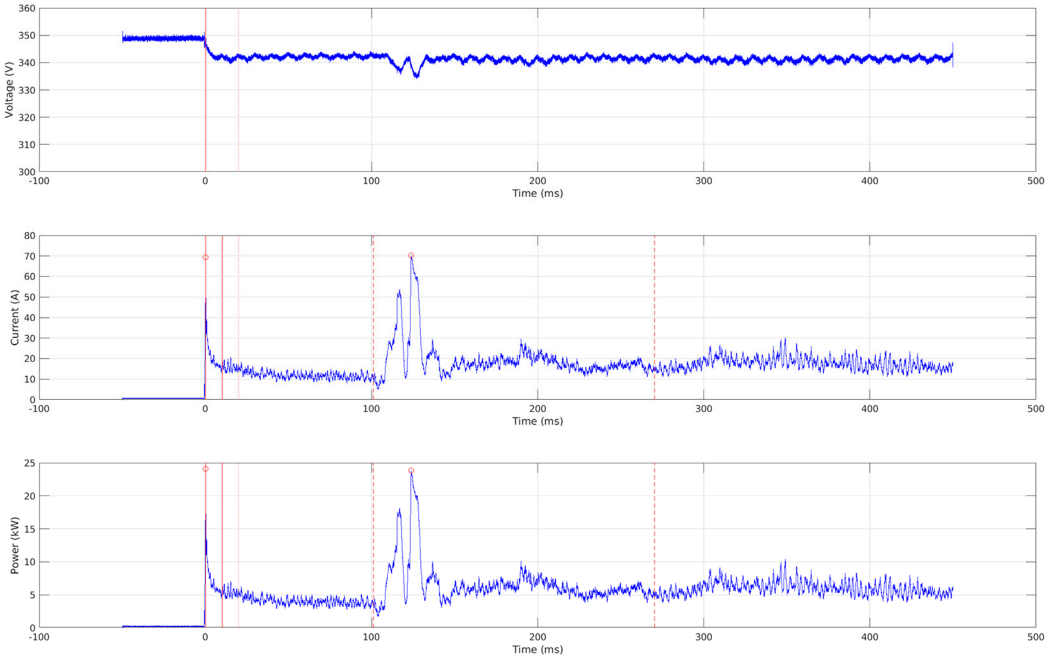

S6: Calculation of the immersion speed that the heat can be effectively conducted to the non-immersed part

The heat transfer rate down a cylindrical part can be described by Fourier's law of heat conduction. For a cylindrical coordinate system, the equation is given by:

$\dot{Q} = -\lambda \cdot S \cdot \frac{\Delta T}{\Delta x}$.

Using Eq 1 provided in the manuscript

$$v \leq \frac{\dot{Q}}{c \cdot \rho \cdot \Delta T \cdot S}$$

and the following values for our example (stainless steel)

$$\text{thermal conductivity} \lambda = 20 \frac{\mathrm{W}}{\mathrm{m} \cdot \mathrm{K}}$$

$$\text{bottom surface} S = 1256 \mathrm{mm}^2$$

$$\text{initial temperature difference between electrolyte temperature and anode temperature} \Delta T = 60 \mathrm{K}$$

$$\text{distance between electrolyte surface and anode top surface} \Delta x = 10 \mathrm{mm}$$

$$\text{specific heat capacity} c = 20 \frac{\mathrm{W}}{\mathrm{m} \cdot \mathrm{K}}$$

$$\text{density} \rho = 8000 \frac{\mathrm{kg}}{\mathrm{m}^3}$$

the immersion velocity *v* should be less than 0.5 mm s^{-1}.

Supporting video
The supporting videos show immersion at all speeds, when the anode is in touch with electrolyte and when it is fully submerged.

Repository
The raw and analysed data, videos, scripts and other data are going to be achieved in Zenodo repository in line with the Data management plan of the Horizon Europe SEAMAC project (GA 101079481).
